# Assessment of serological responses following vaccination campaigns with type 2 novel oral polio vaccine: a population-based study in Tajikistan in 2021

**DOI:** 10.1016/S2214-109X(22)00412-0

**Published:** 2022-11-16

**Authors:** Azamdzhon Mirzoev, Grace R Macklin, Yiting Zhang, Bernardo A Mainou, Umeda Sadykova, Victor Stefan Olsavszky, Shahin Huseynov, Murodali Ruziev, Faizali Saidzoda, Mahtob Bobokhonova, Ondrej Mach

**Affiliations:** a Institute of Postgraduate Education in Health, Dushanbe, Tajikistan; b Polio Eradication Department, World Health Organization, Geneva, Switzerland; c Division of Viral Diseases, Centers for Disease Control and Prevention, Atlanta, GA, USA; d World Health Organization, Country Office, Dushanbe, Tajikistan; e World Health Organization, Regional Office for Europe, Copenhagen, Denmark; f Institute of Preventive Medicine, Dushanbe, Tajikistan; g Republican Center for Immuno-prophylaxis, Dushanbe, Tajikistan

## Abstract

**Background:**

Novel oral poliovirus vaccine type 2 (nOPV2) was used to control an outbreak of type 2 circulating vaccine derived poliovirus (cVDPV2) in Tajikistan, in 2021. We measured seroconversion and seroprevalence of type 2 polio antibodies in children who were reported to have received two doses of nOPV2 in outbreak response campaigns.

**Methods:**

In this community serosurvey, children born after Jan 1, 2016 were enrolled from seven districts in Tajikistan. Dried blood spot cards were collected before nOPV2 campaigns and after the first and second rounds of the campaigns and were sent to the Centers for Disease Control and Prevention (Atlanta, GA, USA) for microneutralisation assay to determine presence of polio antibodies. The primary endpoint was to assess change in seroprevalence and seroconversion against poliovirus serotype 2 after one and two doses of nOPV2.

**Findings:**

228 (97%) of 236 enrolled children were included in the analysis. The type 2 antibody seroprevalence was 26% (53/204; 95% CI 20 to 33) before nOPV2, 77% (161/210; 70 to 82) after one dose of nOPV2, and 83% (174/209; 77 to 88) after two doses of nOPV2. The increase in seroprevalence was statistically significant between baseline and after one nOPV2 dose (51 percentage points [42 to 59], p<0·0001), but not between the first and second doses (6 percentage points [–2 to 15], p=0·12). Seroconversion from the first nOPV2 dose, 67% (89/132; 59 to 75), was significantly greater than that from the second nOPV2 dose, 44% (20/45; 30 to 60; χ^2^ p=0·010). Total seroconversion after two nOPV2 doses was 77% (101/132; 68 to 83).

**Interpretation:**

Our study demonstrated strong immune responses following nOPV2 outbreak response campaigns in Tajikistan. Our results support previous clinical trial data on the generation of poliovirus type 2 immunity by nOPV2 and provide evidence that nOPV2 can be appropriate for the cVDPV2 outbreak response. The licensure and WHO prequalification of nOPV2 should be accelerated to facilitate wider use of the vaccine.

**Funding:**

World Health Organization, Centers for Disease Control and Prevention, and Rotary International.

## Introduction

Substantial progress has been made in the past several years to eradicate wild poliovirus. In 2021, only five cases of poliomyelitis caused by endemic wild poliovirus were detected; four from Afghanistan and one from Pakistan—the last two remaining endemic countries.^1^ Despite this success, in 2021, approximately 691 paralytic cases of poliomyelitis were caused by vaccine-derived polioviruses (VDPVs).^1^

VDPVs result from the use of live Sabin-based oral poliovirus vaccine (OPV), which in rare circumstances regains neurovirulence following prolonged circulation in under-immunised populations.^2^, ^3^, ^4^ Outbreaks of circulating VDPV (cVDPV) continue to be detected in many African and Asian countries, with the vast majority being serotype 2 (cVDPV2).^1^

Response to cVDPV2 outbreaks typically includes vaccination campaigns with OPV2-containing vaccines because inactivated poliovirus vaccines (IPV) induce insufficient intestinal mucosal immunity required to prevent infection and halt virus transmission.^5^ Until October, 2021, Sabin virus-based monovalent OPV2 (mOPV2) has been a vaccine of choice; however, the use of mOPV2 can result in subsequent seeding of new cVDPV2 outbreaks leading to a cycle of repeated cVDPV seeding and repeated vaccination campaigns.^6^ A novel oral poliovirus vaccine type 2 (nOPV2) has been developed to minimise the risk of seeding new cVDPV2 outbreaks.^7^

nOPV2 is a modified version of mOPV2, which was purposefully engineered to be more genetically stable than mOPV2, making it significantly less likely to revert into neurovirulent forms.^8^, ^9^ nOPV2 was authorised under the WHO Emergency Use Listing in November, 2020, exclusively for use in cVDPV2 outbreak response.^10^ Subsequently, Tajikistan was among the first countries in the world to use this vaccine to respond to a cVDPV2 outbreak. nOPV2 showed good safety and immunogenicity in phase 1 and phase 2 trials, but there is no data on the immunogenicity of nOPV2 used in campaign settings.^11^, ^12^, ^13^, ^14^

**Research in context**

**Evidence before this study**

On Nov 13, 2020, a novel live type 2 oral poliovirus vaccine (nOPV2) was recommended for use under WHO Emergency Use Listing (EUL). As part of the clinical development, phase 1 and phase 2 clinical trials have been completed for nOPV2 in Belgium demonstrating the safety, tolerability, and immunogenicity of the vaccine. A larger phase 2 study conducted in Panama confirmed the safety, tolerability, and immunogenicity of nOPV2 in the target population for polio outbreak response in children aged 1–4 years and infants aged 18–22 weeks. Because the authors are part of the research group on nOPV2 development, we did not conduct a formal literature search.

**Added value of this study**

Between Nov 13, 2020, and Oct 14, 2022, approximately 500 million nOPV2 doses have been administered in outbreak response to circulating vaccine-derived type 2 poliovirus (cVDPV2) in 23 countries. This is the first study to provide estimates of immunogenicity of nOPV2 in an outbreak response setting. We observed that vaccination with nOPV2 induced a strong immune response in children younger than 5 years in Tajikistan, in agreement with previous results from phase 1/2 clinical trials. Of note, the first nOPV2 dose resulted in significantly higher seroconversion rates than the second one.

**Implications of all the available evidence**

We demonstrate that high coverage campaigns provide sufficient immunity against cVDPV2 to interrupt transmission, supporting the use of nOPV2 under EUL. Phase 3 trials and longer-term evaluation of safety and genetic stability of nOPV2 are needed to receive full licensure and WHO prequalification for nOPV2.

**Figure 1:**
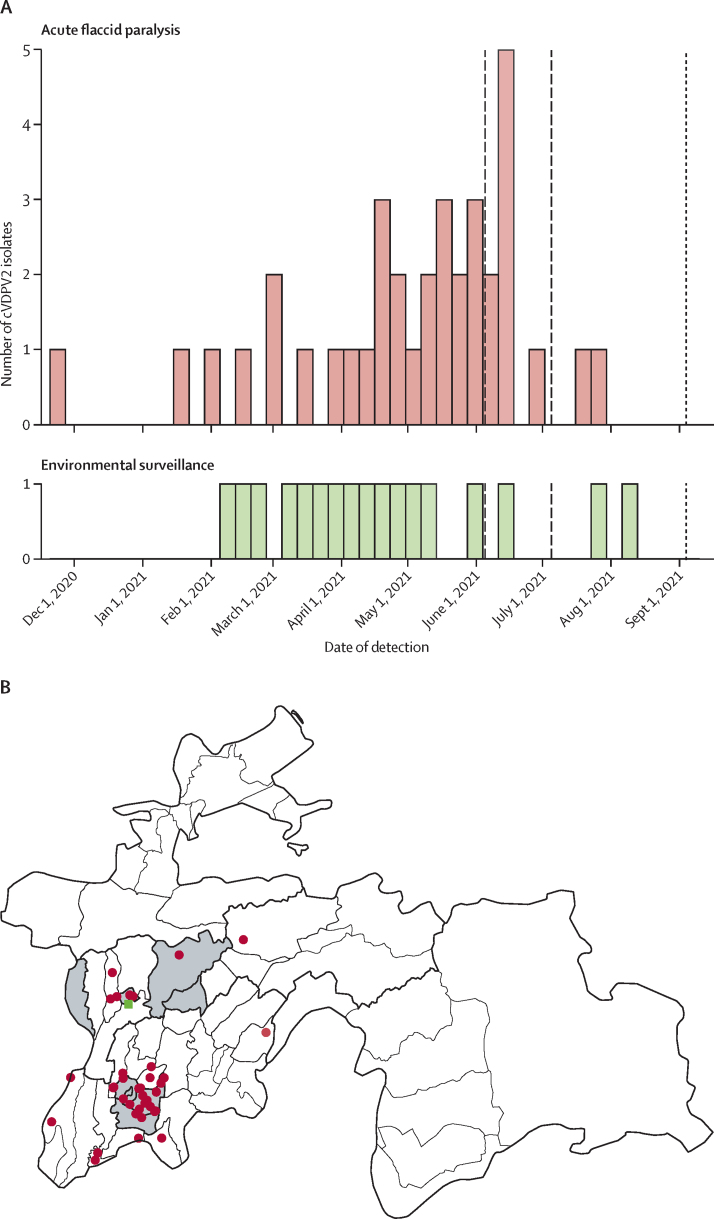
Dates and locations of detection of cVDPV2 isolates in Tajikistan (A) Dates of detection of cVDPV2 isolates in Tajikistan between Nov 1, 2020, and July 31, 2021, from patients with acute flaccid paralysis (n=36) and environmental surveillance samples (n=17). Date of detection represents the date of paralysis onset or date of environmental surveillance sampling. Vertical black lines show the date of nOPV2 outbreak response vaccination campaigns (end date for multi-day campaigns). Dashed lines represent national nOPV2 vaccination campaigns and the dotted line represents the subnational campaign conducted after the timeframe of this study. (B) Location of cVDPV2 detections in Tajikistan from patients with acute flaccid paralysis and environmental surveillance samples, and study areas. Patients with acute flaccid paralysis (red circles), environmental surveillance samples (green squares), and study districts (shaded in grey) are shown. Figure reproduced from data available at WHO headquarters as of July 13, 2021, and reproduced from previous sources.^1^, ^15^ cVDPV2=type 2 circulating vaccine derived poliovirus. nOPV2=novel oral poliovirus vaccine type 2.

In Tajikistan, an outbreak of cVDPV2 was detected in January, 2021. A total of 36 patients with paralytic poliomyelitis have been reported in Tajikistan, with onset of paralysis between Nov 1, 2020, and July 31, 2021 (figure 1).^1^ An environmental surveillance site was subsequently established in Dushanbe with detection of 17 cVDPV2 samples between Feb 1, 2021, and Aug 31, 2021 (figure 1).^15^ Isolates were detected across subnational areas of Districts of Republican Subordination, Khatlon Province, and the capital city Dushanbe (figure 1). The Tajikistan outbreak was genetically linked to a cVDPV2 outbreak first detected in October, 2019, in Pakistan and Afghanistan and was considered as an importation event from one of these countries.^15^, ^16^

The government of Tajikistan responded to the outbreak by conducting a series of vaccination campaigns: initially with IPV in February, 2021, to provide protection against type 2 paralysis in children who had not previously received IPV; followed by two national campaigns of nOPV2 to interrupt cVDPV2 transmission and targeting all children born after Jan 1, 2016, between May 31 and June 5, 2021, and between June 29 and July 5, 2021 (figure 1).^15^ An additional subnational nOPV2 campaign was subsequently conducted due to continued cVDPV2 detection in July and August, 2021, after the completion of our study (figure 1).

In the routine immunisation programme in Tajikistan, children born before April 30, 2016 received trivalent OPV (tOPV; containing all three poliovirus serotypes). From May 1, 2016, the schedule changed to bivalent OPV (bOPV; containing live poliovirus strain serotypes 1 and 3), which does not provide protection against type 2 poliovirus, and was administered at birth and at age 2, 3, 4, and 12 months, and one dose of IPV at age 4 months. IPV introduction into the routine immunisation schedules in Tajikistan was delayed until June 1, 2018, with the IPV catch-up campaign between Feb 15 and 20, 2021, targeting children born between May 1, 2016, and June 30, 2018. WHO/UNICEF estimates that the polio vaccine immunisation coverage in Tajikistan has been consistently higher than 90% in recent years.^17^

To provide rapid evaluation of nOPV2 immunogenicity achieved in vaccination campaign settings, we conducted a serological survey of polio antibodies in children in Tajikistan before and after they received nOPV2 during the two national outbreak response campaigns. The study was conducted in areas that were actively affected by the cVDPV2 outbreak, where exposure to circulating virus could not be controlled.

## Methods

### Study design

This was a community-based, serological survey carried out between May 1 and July 31, 2021. Children born on or after Jan 1, 2016, residing in one of the following districts were eligible for enrolment.

Seven administrative level 2 areas (referred to as districts) were selected for the study, including the capital city Dushanbe (figure 1; appendix p 1). These districts were selected because they were within subnational regions (Districts of Republican Subordination, Khatlon, and Dushanbe) with detection of cVDPV2 isolates. In each district, up to ten health-care facilities were chosen by simple random sampling with Epi Info (version 7) without replacement: Jaloliddin Balkhi (n=1), Dushanbe (n=10), Faizabad (n=3), Kushoniyon (n=1), Tursunzoda (n=1), Vakhdat (n=4), and Vakhsh (n=1; appendix p 1). Children were selected by random selection carried out by district health immunisation directors based on available lists of children using health records in each of the selected health-care facilities.

The study received ethical clearance from WHO (ERC.0003599) and the Committee of Biomedical Ethics, Ministry of Health and Social Protection of the Population, Tajikistan.

### Procedures

The study procedures were carried out during three health centre visits. Visit 1 was in the days before the first nOPV2 campaign, visit 2 was 1 month after the first campaign of nOPV2 concluded (just before the second nOPV2 campaign), and visit 3 was 1 month after the second campaign concluded. At the first visit, after obtaining written informed consent from the child's parents or guardians, children were enrolled, and a simple demographic questionnaire was taken, which included age, gender, and poliovirus vaccination history; vaccination history of IPV (through routine immunisation or catch-up campaign) and bOPV (through routine immunisation) was recorded through vaccination cards where available, or parental recall. At the second and third visits, inclusion in the preceding nOPV2 campaign was recorded through parental recall. Trained phlebotomists generated dried blood spots (DBSs) on Whatman 903tm cards using a finger prick technique at each of the three health centre visits for each child. A third subnational nOPV2 campaign was conducted after the last visit, due to continued detection of cVDPV2, but this campaign did not affect the analysis in this study because it occurred after all blood samples were collected and the timeframe was outside of the approved protocol.

The DBS cards were sent to the Centers for Disease Control and Prevention in Atlanta, GA, USA and were tested for the presence of poliovirus neutralising antibodies using standard microneutralisation assays.^18^ The maximum dilution of samples tested was 1/1024 (and highest detectable titre reported was 1/1448 or more); the minimum (non-detectable) titre reported was less than 1/8.

### Outcomes

The primary endpoint of the study was to assess change in seroprevalence and seroconversion against poliovirus serotype 2 after one and two doses of nOPV2 administered as part of outbreak response campaigns. The secondary endpoint was to describe seroprevalence against poliovirus serotypes 1 and 3.

Seropositivity for each serotype was defined as the reciprocal titre of poliovirus neutralising antibodies of 8 or more. Seroconversion was defined as the change from seronegative to seropositive (from reciprocal titre of <8 to ≥8) in children with antibodies at baseline. Boosting was defined as at least a 4 times increase in reciprocal titres in children that were seropositive at the initial timepoint of comparison. Analyses of seroconversion and boosting are both restricted to children aged 6 months or older at enrolment (to avoid bias caused by maternal antibody interference); boosting analysis was further restricted to children with initial antibody titres of 362 or less to show a 4 times increase given the maximum reported titre of 1/1448.

### Statistical analysis

The total sample size was calculated to be 215 children. We assumed a 30% dropout rate during follow-up (estimated higher due to the COVID-19 pandemic) and laboratory analysis and a baseline seroprevalence of 40% to provide 90% power to detect a change in seroprevalence of at least 15%.

Seroprevalence, seroconversion, and boosting are presented in percentages with binomial 95% CIs. Median antibody titres are provided with IQR. Pearson's χ^2^ test is used to determine if there was a statistically significant difference between seroprevalence across different timepoints. For univariate risk analysis of seroconversion, binomial generalised linear models were generated to calculate statistical significance.^19^ All analysis was conducted using R (v4.2.2) statistical computing software.

### Role of the funding source

WHO employees participated in the study design, data collection, data analysis, data interpretation, and writing of the report.

## Results

**Figure 2:**
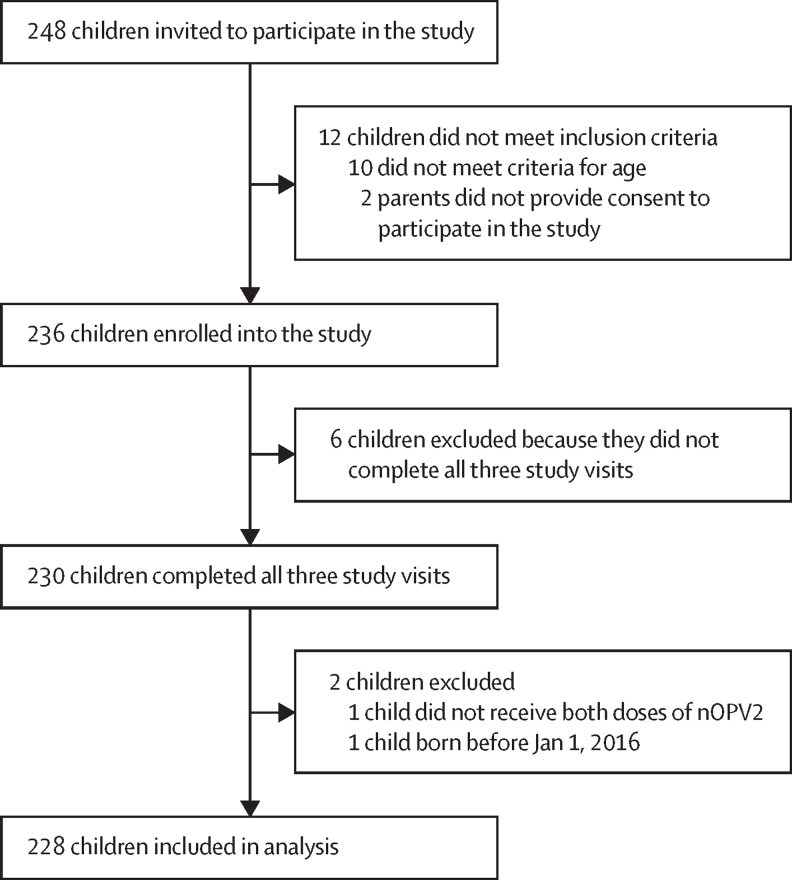
Study participant flow diagram nOPV2=novel oral poliovirus vaccine type 2.

Of the 248 children invited to participate in the study, 236 (95**%**) were enrolled (figure 2). The remaining children either did not meet inclusion criteria for age (n=10) or their parents did not provide consent to participate in the study (n=2). All three study visits were completed by 230 (97%) of the 236 enrolled children. The six children that did not complete all three study visits were excluded. 229 of the children that completed all three study visits were reported to have received both nOPV2 doses as part of the outbreak response campaigns. One child did not receive both doses and was excluded from the analysis. One additional child was excluded after enrolment because they were born before Jan 1, 2016. Therefore, 228 (97%) of the 236 enrolled children were included in the analysis. Out of the 684 DBS samples from the 228 children in the analysis, 648 (95%) had sufficient blood for testing against serotype 1, 623 (91%) had sufficient blood for testing against serotype 2, and 642 (94%) had sufficient blood for testing against serotype 3.

The median age of participants was 36 months (range 2–66) at enrolment, with 19 (8%) of 228 children born between Jan 1 and April 30, 2016, who received tOPV instead of bOPV in their routine immunisation (table 1). 222 (97%) of 228 children had a vaccination card available and 200 (88%) of 228 children were reported as receiving at least one dose of IPV either as part of routine immunisation or during the IPV campaign in February, 2021.

**Table 1:** Demographic indicators and vaccination history

		**Children included in analysis (n=228)**
Gender		
	Female	112 (49%)
	Male	116 (51%)
Median age at enrolment, months		36 (2–66)
Age at enrolment		
	0–6 months	6 (3%)
	7–12 months	18 (8%)
	13–36 months	92 (40%)
	37–61 months	93 (41%)
	62–66 months*	19 (8%)
Administrative 2 area (district)		
	Dushanbe	56 (25%)
	Faizabad	30 (13%)
	Tursunzoda	30 (13%)
	Vakhdat	30 (13%)
	Kushoniyon	26 (11%)
	Vakhsh	29 (13%)
	Jaloliddin Balkhi	27 (12%)
Available vaccination card		222 (97%)
Inactivated poliovirus vaccine received, either in routine immunisation or catch-up campaign		200 (88%)
Median number of bivalent OPV doses received via routine immunisation		5 (1–5)
Bivalent OPV doses received, routine immunisation		
	1 dose	3 (1%)
	2 doses	1 (<1%)
	≥3 doses	221 (97%)
	Unknown	3 (1%)

Data are n (%) or median (range). OPV=oral poliovirus vaccine.

* Born before the switch from trivalent OPV to bivalent OPV (Jan 1–April 30, 2016).

**Figure 3:**
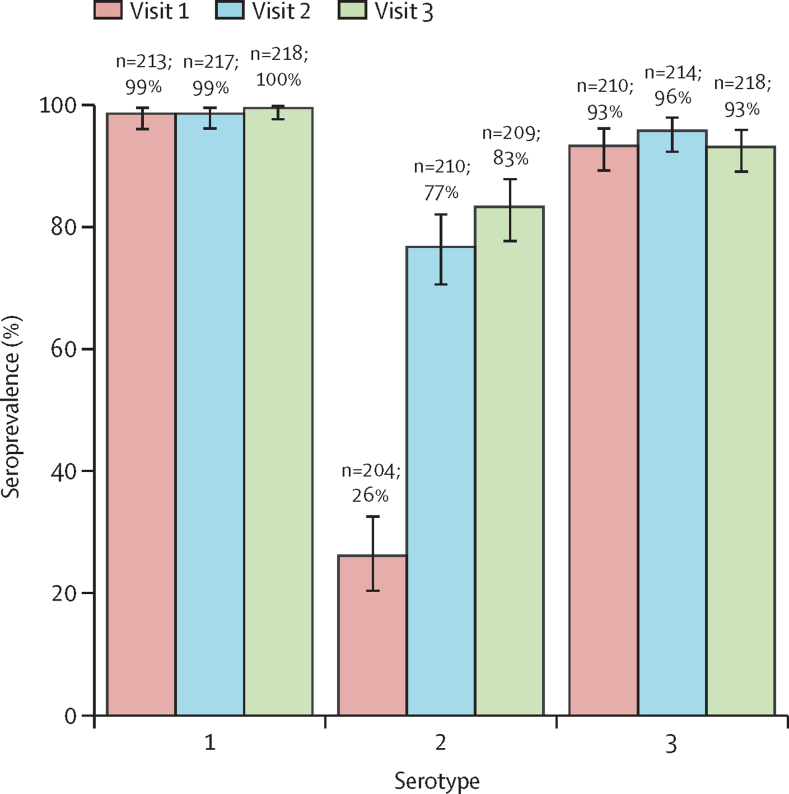
Seroprevalence of poliovirus serotype 1, 2, and 3 neutralising antibodies before nOPV2 campaigns (visit 1), 1 month after the first nOPV2 campaign (visit 2), and 1 month after the second nOPV2 campaign (visit 3) 95% binomial CIs indicated by error bars, and sample size of analysable samples and percentage are shown. nOPV2=novel oral poliovirus vaccine type 2.

At visit 1 (baseline visit), 53 of 204 children (26% [95% CI 20–33]) with analysable samples were seropositive against poliovirus type 2 (figure 3). Baseline seroprevalence was significantly higher in children born before the switch from tOPV to bOPV (Jan 1–April 30, 2016), 63% (95% CI 38–84; 12/19), than in children born after the switch from tOPV to bOPV, 22% (16–29; 41/185; p<0·001; appendix pp 2–3). In children born after the switch from tOPV to bOPV, baseline seroprevalence was not significantly different between children who were reported as receiving IPV, 22% (16–29; 36/163), and children who were reported as not receiving IPV, 23% (8–45; 5/22; p=0·95).

After one dose of nOPV2 the seroprevalence against type 2 poliovirus increased to 77% (95% CI 70 to 82; 161/210) and after two doses of nOPV2 the seroprevalence against type 2 poliovirus increased to 83% (77 to 88; 174/209; figure 3). This increase in seroprevalence was statistically significant between baseline and after one nOPV2 dose (51 percentage points [42 to 59]; p<0·0001), but not between the first and second doses (6 percentage points [–2 to 15]; p=0·12).

**Figure 4:**
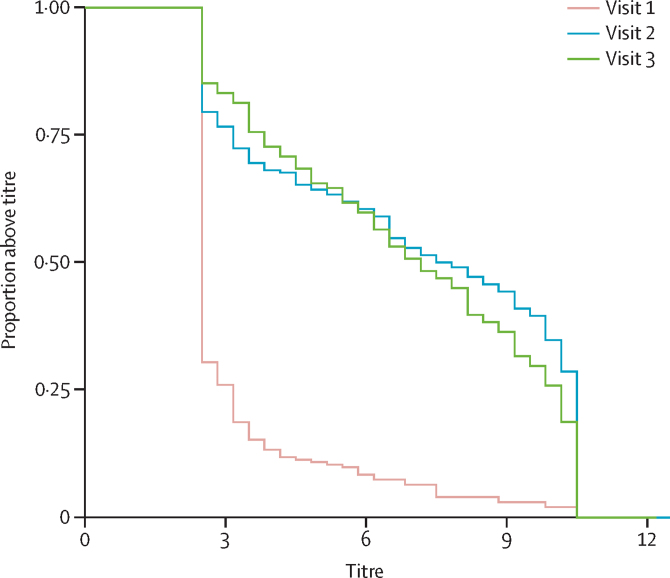
Reverse cumulative distribution curves of antibody titres for poliovirus serotype 2 before nOPV2 campaigns (visit 1), 1 month after the first nOPV2 campaign (visit 2), and 1 month after the second nOPV2 campaign (visit 3) Titres on x axis expressed in log_2_ scale. Antibody titres range from 2·50 to 10·50 log_2_. nOPV2=novel oral poliovirus vaccine type 2.

The reverse cumulative antibody titres against serotype 2 at each of three visits are shown in figure 4. Median reciprocal antibody titre on a log_2_ scale was 2·50 (IQR 2·50–3·17) at visit 1, 7·77 (3·17–10·50) at visit 2; and 7·17 (3·83–10·17) at visit 3. The limits of detection of the assay are 2·50 log_2_ and 10·50 log_2_.

Rates of seroconversion were calculated for children older than 6 months with three analysable samples (n=182). The proportion of children that seroconverted between visit 1 and 2 was 67% (89/132; 95% CI 59–75), between visit 2 and 3 was 44% (20/45; 30–60), and between visit 1 and 3 was 77% (101/132**;** 68–83; table 2). The first nOPV2 dose was more immunogenic than the second (seroconversion 67% *vs* 44%; p= 0·010). The proportion of children that were boosted between visit 1 and 2 was 58% (29/50; 43–72); between visit 2 and 3 was 9% (12/137; 5–15), and between visit 1 and 3 was 54% (27/50; 39–68).

**Table 2:** Seroconversion and boosting of type 2 antibodies after one dose and two doses of nOPV2

	**Number of children**
**Seroconversion**	
Visit 1 to visit 2; nOPV2 dose 1	89/132; 67% (59–75)
Visit 2 to visit 3; nOPV2 dose 2	20/45; 44% (30–60)
Visit 1 to visit 3; nOPV2 dose 1 and 2	101/132; 77% (68–83)
**Boosting**	
Visit 1 to visit 2; nOPV2 dose 1	29/50; 58% (43–72)
Visit 2 to visit 3; nOPV2 dose 2	12/137; 9% (5–15)
Visit 1 to visit 3; nOPV2 dose 1 and 2	27/50; 54% (39–68)

Data are n/N; % (95% CI). Analysis was restricted to children older than 6 months with three sufficient dry blood spot samples (n=182). Seroconversion analysis is restricted to individuals that are seronegative at the baseline timepoint of comparison. Boosting analysis is restricted to individuals that are seropositive at the baseline timepoint of comparison (individuals that seroconvert to the first dose become eligible for boosting analysis for the second dose). nOPV2=novel oral poliovirus vaccine type 2.

We conducted an analysis of risk factors for type 2 serological non-response; in this analysis we included children who completed all three visits and provided three analysable samples and who were seronegative for type 2 (n=132). There was no significant difference in the rate of seroconversion between boys (72%; 50/69) and girls (81%; 51/63; p=0·25) or in children who were reported as receiving a dose of IPV (80%; 12/15) or those who did not receive IPV (76%; 89/117; p=0·74; table 3). Additionally, there was no significant difference in seroconversion for the different age groups (p>0·1 for all age groups) compared with baseline of 67% (6/9) in the 7–12 months age group. The seroconversion varied across the seven districts, with the highest in Dushanbe at 90% (28/31).

**Table 3:** Univariate analysis of factors associated with seroconversion for poliovirus serotype 2 after two doses of nOPV2 (between visit 1 and visit 3) in children older than 6 months, with three analysable type 2 poliovirus samples, and seronegative at visit 1 (baseline; n=132)

	**Seroconversion**	**p value, binomial generalised linear model**
**Gender**		
Male	72% (50/69)	Ref
Female	81% (51/63)	0·25
**Administrative 2 area (district)**		
Dushanbe	90% (28/31)	Ref
Faizabad	73% (16/22)	0·11
Tursunzoda	71% (10/14)	0·12
Vakhdat	82% (9/11)	0·46
Kushoniyon	78% (14/18)	0·24
Vakhsh	67% (12/18)	0·048
Jaloliddin Balkhi	67% (12/18)	0·048
**Inactivated poliovirus vaccine received**		
No	76% (89/117)	Ref
Yes	80% (12/15)	0·74
**Age at enrolment**		
7–12 months	67% (6/9)	Ref
13–36 months	69% (40/58)	0·89
37–61 months	88% (52/59)	0·11
62–66 months	50% (3/6)	0·52

Data are % (n/N) unless otherwise stated.

Seroprevalence against serotypes 1 and 3 was more than 90% (figure 4). There was no significant difference between the seroprevalence at any timepoint for either type 1 (p>0·97) or type 3 (p>0·99).

## Discussion

This was the first study to provide estimates of immunogenicity of nOPV2 administered in outbreak response campaigns. We observed robust immune response to nOPV2 in agreement with previous results from phase 1/2 clinical trials:^13^ type 2 seroconversion after one dose was 67% and after two doses it was 77%, and type 2 seroprevalence increased from 26% at baseline to 83% after two doses.

In our study, we observed that the first nOPV2 dose resulted in significantly higher seroconversion rates than the second dose (67% *vs* 44%, p=0·010). We hypothesise that some children had been immunologically primed by one IPV dose but had not seroconverted and that these children readily responded to the first nOPV2 dose.^20^, ^21^ It is also possible that the ongoing background circulation of cVDPV2 continued after the first nOPV2 dose but was reduced or eliminated after the second dose and that this affected seroconversion rates in the period when the first dose was administered. Antibody titres significantly increased after the first nOPV2 dose but remained relatively unchanged after the second dose was administered.

Baseline seroprevalence against type 2 was low (26%) and does not correspond with the reported IPV coverage in our study (88%), or with WHO/UNICEF estimates (97%). Type 2 seroconversion after a bOPV schedule with a single dose of IPV administered at 3–4 months has been measured between 50% and 80%; therefore, our results suggest that IPV in Tajikistan achieved a lower seroconversion or that the coverage was overestimated.^22^, ^23^, ^24^, ^25^, ^26^ The 26% baseline seroprevalence indicated that our study was not underpowered as the assumption was 40%, which resulted in a larger sample size than needed.

We observed that seroconversion rates were higher in the capital city Dushanbe (>90%) than in other districts (around 66%) and that this difference was borderline significant (p=0·048). This difference could indicate that the cVDPV2 circulation in Dushanbe was more intense than in the other areas surveyed, resulting in higher seroconversion in Dushanbe from a combination of the nOPV2 vaccine and natural infection with cVDPV2. The environmental surveillance site in Dushanbe consistently detected positive samples for cVDPV2 during the study period; however, environmental surveillance was not established elsewhere for comparison. Demographic or health disparities (such as prevalence of enteric pathogens) between the capital city and other districts that affect vaccine performance could also be considered.^27^

We demonstrated uniformly high seroprevalence against poliovirus types 1 and 3, indicating successful implementation of routine immunisation with bOPV in Tajikistan (there have been no vaccination campaigns with bOPV since it was introduced in April, 2016). There were also no significant differences in type 1 and 3 seroprevalence rates following nOPV2 vaccination as expected.

Our study had some limitations. The study was conducted under extraordinary circumstances in Tajikistan during a peak of the COVID-19 pandemic and active poliovirus outbreak, which affected availability of health-care personnel for this study. The study used simple random sampling and was conducted in areas that were affected by the active cVDPV2 outbreak; therefore, it is possible that individuals might have serologically converted in response to infection with cVDPV2. Although no cases of acute flaccid paralysis were reported in study participants, the study did not monitor shedding of cVDPV2 in participants or household contacts; however, evidence suggests that in areas with active transmission, only a small proportion of children in the community excrete poliovirus at any time.^28^ Further, IPV vaccination history was not consistently recorded, making it challenging to establish if IPV was received from routine immunisation or through the catch-up campaign in February, 2021.

The cVDPV2 outbreak in Tajikistan has been officially closed by WHO and the Tajik Ministry of Health, with the last detected isolate in an environmental sample from Dushanbe on Aug 13, 2021. Our study demonstrates that high coverage campaigns with nOPV2 provide an immune response against cVDPV2 and probably interrupted transmission in Tajikistan, providing evidence that nOPV2 vaccine is an appropriate tool to interrupt cVDPV2 transmission during outbreaks. Phase 3 clinical trials and long-term evaluation of safety and genetic stability of nOPV2 need to be performed, to enable consideration of full prequalification from WHO for the nOPV2 vaccine.

## Data sharing

Individual participant data will not be made publicly available. Contact the corresponding author for inquiries regarding data.

## Declaration of interests

We declare no competing interests.
