# Supplementary material for: Assessment of serological responses following vaccination campaigns with type 2 novel oral polio vaccine: a population-based study in Tajikistan in 2021
Source: Lancet Glob Health. 2022 Nov 16;10(12):e1807–14. doi: 10.1016/S2214-109X(22)00412-0 (PMC9681660; doi:10.1016/S2214-109X(22)00412-0)
Supplement: Supplementary appendix [file mmc1.pdf]

THE LANCET
Global Health

Supplementary appendix

This appendix formed part of the original submission and has been peer reviewed. We post it as supplied by the authors.

Supplement to: Mirzoev A, Macklin GR, Zhang Y, et al. Assessment of serological responses following vaccination campaigns with type 2 novel oral polio vaccine: a population-based study in Tajikistan in 2021. *Lancet Glob Health* 2022; **10:** e1807–14.

Supplementary Table 1: Demographics and primary healthcare facilities in districts selected for study

Administrative 1 Area (Region)	Administrative 2 Area (District)	Number of primary healthcare facilities selected for study	Total number of primary healthcare facilities providing immunization services	Under 5 population size
Dushanbe	Dushanbe	10	55	125,063
Districts of Republican Subordination	Tursunzoda	1	80	41,945
Districts of Republican Subordination	Vahdat	4	111	48,352
Districts of Republican Subordination	Faizabad	3	45	14,746
Khatlon	Kushoniyon	1	76	33,387
Khatlon	Vakhsh	1	69	28,351
Khatlon	Jaloliddin Balkhi	1	57	29,350

Supplementary Table 2: Seroprevalence by visit and age group to poliovirus types 1, 2 and 3.

Age at enrollment (months)	Visit	Serotype	Number of individuals with analyzable samples	Number seropositive	Seroprevalence (%)	95% Confidence Intervals (%)
0-6	1	1	6	6	100	54.1, 100
7-12	1	1	16	16	100	79.4, 100
13-36	1	1	83	82	98.8	93.5, 100
37-61	1	1	89	87	97.8	92.1, 99.7
62-66	1	1	19	19	100	82.4, 100
**Total**	**1**	**1**	**213**	**210**	**98.6**	**95.9, 99.7**
0-6	1	2	6	2	33.3	4.3, 77.7
7-12	1	2	14	4	28.6	8.4, 58.1
13-36	1	2	78	12	15.4	8.2, 25.3
37-61	1	2	87	23	26.4	17.6, 37
62-66	1	2	19	12	63.2	38.4, 83.7
**Total**	**1**	**2**	**204**	**53**	**26.0**	**20.1, 32.6**
0-6	1	3	6	2	33.3	4.3, 77.7
7-12	1	3	16	15	93.8	69.8, 99.8
13-36	1	3	82	76	92.7	84.8, 97.3
37-61	1	3	87	84	96.6	90.3, 99.3
62-66	1	3	19	19	100	82.4, 100
**Total**	**1**	**3**	**210**	**196**	**93.3**	**89.1, 96.3**
0-6	2	1	6	6	100	54.1, 100
7-12	2	1	16	16	100	79.4, 100
13-36	2	1	84	83	98.8	93.5, 100
37-61	2	1	92	90	97.8	92.4, 99.7
62-66	2	1	19	19	100	82.4, 100
**Total**	**2**	**1**	**217**	**214**	**98.6**	**96, 99.7**
0-6	2	2	6	6	100	54.1, 100
7-12	2	2	15	10	66.7	38.4, 88.2

13-36	2	2	79	56	70.9	59.6, 80.6
37-61	2	2	91	73	80.2	70.6, 87.8
62-66	2	2	19	16	84.2	60.4, 96.6
**Total**	**2**	**2**	**210**	**161**	**76.7**	**70.4, 82.2**
0-6	2	3	6	4	66.7	22.3, 95.7
7-12	2	3	16	16	100	79.4, 100
13-36	2	3	82	80	97.6	91.5, 99.7
37-61	2	3	91	87	95.6	89.1, 98.8
62-66	2	3	19	18	94.7	74, 99.9
**Total**	**2**	**3**	**214**	**205**	**95.8**	**92.2, 98.1**
0-6	3	1	6	6	100	54.1, 100
7-12	3	1	18	18	100	81.5, 100
13-36	3	1	87	87	100	95.8, 100
37-61	3	1	89	88	98.9	93.9, 100
62-66	3	1	18	18	100	81.5, 100
**Total**	**3**	**1**	**218**	**217**	**99.5**	**97.5, 100**
0-6	3	2	6	6	100	54.1, 100
7-12	3	2	18	13	72.2	46.5, 90.3
13-36	3	2	81	62	76.5	65.8, 85.2
37-61	3	2	86	78	90.7	82.5, 95.9
62-66	3	2	18	15	83.3	58.6, 96.4
**Total**	**3**	**2**	**209**	**174**	**83.3**	**77.5, 88**
0-6	3	3	6	4	66.7	22.3, 95.7
7-12	3	3	18	17	94.4	72.7, 99.9
13-36	3	3	87	82	94.3	87.1, 98.1
37-61	3	3	89	82	92.1	84.5, 96.8
62-66	3	3	18	18	100	81.5, 100
**Total**	**3**	**3**	**218**	**203**	**93.1**	**88.9, 96.1**
